# The Central Role of Hypothermia and Hyperactivity in Anorexia Nervosa: A Hypothesis

**DOI:** 10.3389/fnbeh.2021.700645

**Published:** 2021-08-06

**Authors:** Lucille Lakier Smith

**Affiliations:** Human Performance Laboratory, Department of Kinesiology, School of Health Sciences, East Carolina University, Greenville, NC, United States

**Keywords:** core temperature (T_core_), skin temperature (T_skin_), thermo-behavior, hyperactivity (HyAc), excessive exercise (ExEx), non-exercise activity thermogenesis (NEAT), spontaneous physical activity (SPA)

## Abstract

Typically, the development of anorexia nervosa (AN) is attributed to psycho-social causes. Several researchers have recently challenged this view and suggested that *hypothermia and hyperactivity* (HyAc) are central to AN. The following hypothesis will attempt to clarify their role in AN. Anorexia nervosa patients (ANs) have significantly lower core temperatures (T_core_) compared to healthy controls (HCs). This reduced temperature represents a reset T_core_ that needs to be maintained. However, ANs cannot maintain this T_core_ due primarily to a reduced basal metabolic rate (BMR); BMR usually supplies heat to sustain T_core_. Therefore, to generate the requisite heat, ANs revert to the *behavioral-thermoregulatory strategy* of *HyAc*. The majority of ANs (~89%) are reportedly HyAc. Surprisingly, engagement in HyAc is *not* motivated by a conscious awareness of low T_core_, but rather by the innocuous sensation of “cold- hands” frequently reported by ANs. That is, local hand-thermoreceptors signal the brain to initiate HyAc, which boosts perfusion of the hands and alters the sensation of “cold-discomfort” to one of “comfort.” This “rewarding” consequence encourages repetition/habit formation. Simultaneously, hyperactivity increases the availability of heat to assist with the preservation of T_core_. Additionally, HyAc induces the synthesis of specific *brain neuromodulators* that *suppress food* intake and further *promote* HyAc; this outcome helps preserve low weight and perpetuates this vicious cycle. Based on this hypothesis and supported by rodent research, external heat availability should reduce the compulsion to be HyAc to thermoregulate. A reduction in HyAc should *decrease the production* of brain neuromodulators that suppress appetite. If verified, hopefully, this hypothesis will assist with the development of novel treatments to aid in the resolution of this intractable condition.

## Introduction

In 1868, Gull (1997) published the first medical report on anorexia nervosa (AN). This publication dealt with the classic symptoms of abnormally low body weight and refusal to eat, predominantly in female patients. It also focused on the role of hypothermia and hyperactivity in AN. Over the last three decades, the importance of hyperactivity in AN is reflected in the publication of over 400 articles and seven reviews (Rizk et al., 2020). Additionally, numerous research articles have focused on the role of hypothermia in AN.

During the 20th century, many professionals assumed that a mental/psychological proclivity, such as obsessive-compulsive disorder or anxiety-disorder, *initiated* the drastic weight loss associated with AN (Sodersten et al., 2019a). For this reason, research and treatment strategies for AN were subsumed under the discipline of psychology. However, many psychologists/psychiatrists presently consider the available treatments for ANs, including psychotherapy and pharmacotherapy, inadequate (Kaye, 2008; Bergh et al., 2013; Sodersten et al., 2019b; Gutierrez and Birmingham, 2020). Recently, it has been suggested that the “future development of a treatment for AN should explore new routes as the current conceptualization of AN seems to be on the wrong track” (Gutierrez and Carrera, 2018).

Although most agree that most anorexia nervosa patients (ANs) regularly engage in excessive amounts of exercise, this behavior i*s classified as a secondary symptom in diagnosis* (Gutierrez and Birmingham, 2020; Rizk et al., 2020). Another symptom overlooked in the AN diagnosis is hypothermia, a low core temperature (T_core_; Swenne and Engstrom, 2005).

A common belief is that HyAc is a strategy deliberately employed by ANs to burn calories (Dittmer et al., 2018). However, Gutierrez and Vazquez (2001) have proposed that HyAc, which *hinders* recovery, develops in response to a low T_core_. This observation led them to propose that a supply of heat could help neutralize HyAc, and in so doing, help rehabilitate the anorexic patient (ANs). The present author supports this premise.

The primary objective of this article is to define the underlying mechanism of hypothermia and HyAc in AN. Hopefully, this will stimulate research to either support, modify, or refute this hypothesis.

## Anorexia Nervosa

While dieting behavior is almost universal, especially in cultures with an emphasis on thinness, the condition of AN may occur wherein an individual willfully self-starves in the presence of plentiful food. This chronic disorder has an incidence rate of 0.3–0.9%, with 90% of the afflicted being females (Yilmaz et al., 2015). Furthermore, the overall mortality rate for AN is higher than any other psychiatric disorder (Walsh, 2013; Misra and Klibanski, 2014; Yilmaz et al., 2015; Nagata et al., 2017; Casper, 2020).

A salient feature is the initial dramatic weight loss and the subsequent maintenance of this abnormally low weight. ANs have less than 85% of expected body weight and a body mass index (BMI) less than 17.5 kg/m^2^. This condition should be differentiated from constitutionally lean, healthy individuals who also have low body weight and BMI but do not have an eating disorder (Estour et al., 2017).

ANs rarely have complete suppression of appetite. Instead, they exhibit extreme resistance to feeding drives (Casper, 1998) while simultaneously being preoccupied with food and eating rituals (Kaye, 2008). They also deny being underweight, refuse to accept the seriousness of the medical consequences (Casper, 1998), and usually resist treatment (Kaye, 2008). They have other clusters of puzzling symptoms, including excessive exercise (Kaye, 2008).

The origins of disturbed eating behaviors are poorly understood. Over the last few decades, beliefs about the causes of AN have undergone extensive changes (Beumont et al., 1987). It now appears that eating disorders aggregate in families; twin studies reveal that additive genetic factors account for approximately 40–60% of AN (Trace et al., 2013). AN is now viewed as a complex disorder resulting from a combination of genetic and environmental factors (Yilmaz et al., 2015).

## Anorexia Nervosa and Hypothermia

Gull (1997), who coined the term “anorexia nervosa,” noted that many of his extremely emaciated female patients had a body core temperature (T_core_) below the normal 37°C. Research has since confirmed that ANs are mildly hypothermic, having a T_core_ significantly lower than healthy controls (HCs; ~0.6°C–2°C; Wakeling and Russell, 1970; Lampert and Lau, 1976; Fohlin, 1977; Davies et al., 1979; Luck and Wakeling, 1980; Misra and Klibanski, 2014; Chudecka and Lubkowska, 2016). Furthermore, Gull (1997) reported that these patients constantly complained of “feeling cold” and especially of having “cold hands.” This condition has also been confirmed (Crisp, 1967; Wakeling and Russell, 1970; Lampert and Lau, 1976; Swenne and Engstrom, 2005; Kurklinsky et al., 2011; Das and Maiti, 2013).

Although several AN-related hypotheses have been proposed to explain this mild hypothermia, as well as reports of “feeling cold/cold hands” (Wakeling and Russell, 1970; Mecklenburg et al., 1974; Luck and Wakeling, 1980; Chudecka and Lubkowska, 2016), none have been substantiated (Davies et al., 1979). A reasonable assumption is that the frequent complaints of “feeling cold/cold hands” results from the lower T_core_, suggesting a causal relationship between these two phenomena (Luck and Wakeling, 1980). It will be argued that, for the most part, this is *not* the case (Davies et al., 1979). An alternate hypothesis will now be presented:

- Regarding the **lower T_core_**, it will be *suggested* that:
  - T_core_ *is reset* at a lower level.
  - This reset T_core_ is a regulated physiological response to weight-loss/caloric restriction (Taylor and Keys, 1950; Landsberg, 2012).
  - This lower set-point is the “new normal” for ANs.
  - This lower *set-point* must now be maintained/defended.
- Concerning ANs frequent complaints of “feeling cold” and especially “cold-hands,” it will be suggested that the *overall* sensation of “chilliness” is a result of cold sensations registered in the hands and not directly related to the low T_core_. Furthermore, these cold-hand sensations motivate engagement in “corrective” heat-seeking thermo-behavior.

Reduced T_core_ will now be addressed.

## Downward Resetting of Core Temperature

The maintenance of T_core_ is a crucial homeostatic function. Normal-weight, healthy individuals maintain a T_core_ of 37°C within a few tenths of a degree centigrade (~0.2°C), even at different ambient temperatures (Romanovsky, 2007; Filingeri, 2016; Schlader and Vargas, 2019). Any deviation from this homeostatic level activates various involuntary responses, such as shivering, to safeguard T_core_.

T_core_ of 37°C is generally higher than ambient room temperature; a comfortable room temperature is ~23°C. Under ordinary circumstances, HCs can maintain T_core_ even though T_core_ is ~14°C higher than room temperature. This is achieved by having a balance between two coordinated reactions: the amount of heat *retained within* the body (vasoconstriction and insulation) and the amount of heat *generated* within the body (metabolic rate; Landsberg, 2012).

Although HCs can attain this balance, ANs are unable to achieve this (Lampert and Lau, 1976; Luck and Wakeling, 1980; Misra and Klibanski, 2014; Chudecka and Lubkowska, 2016). It will now be proposed that in ANs, a combination of *greater heat loss to the environment* (Mecklenburg et al., 1974; Chudecka and Lubkowska, 2016), as well as *lower production of metabolic heat* (Casper et al., 1991; Onur et al., 2005) is associated with the *resetting of T_core_* to a lower level.

### Greater Loss of Body Heat to the Environment

#### Reduced Vasoconstriction

HCs depend on the sympathetic nervous system (SNS) to achieve thermal balance (Landsberg, 2012). The SNS, the primary innovator of the vascular system, activates both vasoconstriction and vasodilation (Landsberg, 2012). Thus, in HCs, if T_core_ “threatens” to fall below 37°C, blood vessels contract (vasoconstriction), reducing blood flow and associated heat to the periphery and thus preserving this metabolic heat for T_core_ (Brychta and Chen, 2017).

Loss of body heat may be assessed indirectly by measuring body surface temperatures. In ANs, due to the low T_core_, one would intuitively anticipate increased vasoconstriction to retain body heat (Freyschuss et al., 1978). *Surprisingly, this is not the case*! Chudecka and Lubkowska (2016) used thermography to assess the average skin temperatures (T_skin_) of 12 different body areas, using 15 female ANs (BMI of 15.58 kg/m^2^), and compared this to 100 female HCs (BMI of 21.74 kg/m^2^). There were no significant differences between the two groups in the following four areas: chest, front, and back of the forearm and the front of the shank. Surprisingly, in six other body areas, which represented most of the body’s surface area (upper back, lower back, abdomen, front and back of the thigh, and back of the shank), T_skin_ was *significantly higher in AN* compared to HCs. These elevated skin temperatures in ANs, over most body areas, imply *reduced* vasoconstriction (Mecklenburg et al., 1974) and increased heat loss to the environment.

Reduced body surface vasoconstriction, reflective of *reduced SNS activation*, is assessed indirectly by measuring norepinephrine (NE) levels in plasma, cerebrospinal fluid (CSF), and/or urine (Young and Landsberg, 1977a). Pirke (1996) reviewed the evidence of NE responses in ANs. He concluded that “All results indicate reduced noradrenergic activity in the central and peripheral nervous system of patients with eating disorders.” The notion that SNS is suppressed during severe caloric restriction is supported by others (Young and Landsberg, 1977b).

Additional evidence of reduced SNS activation in ANs, is provided by measuring heart rate variability (HRV). HRV is the beat-by-beat variance in heart rate and is primarily an indicator of cardiac autonomic tone. Generally, the higher the HRV, the greater the parasympathetic activation compared to sympathetic activation. Patients with AN have markedly and consistently elevated HRV compared to controls and young athletes, thus supporting the role of reduced SNS activity in AN (Peyser et al., 2021).

Pirke (1996) has suggested that the clinical consequences of reduced SNS activity include hypothermia, hypotension, and bradycardia, as well as reduced peripheral perfusion, all factors evident in ANs (Freyschuss et al., 1978; Luck and Wakeling, 1981).

#### Reduced Body Insulation

Subcutaneous adipose tissue is an effective insulator, which generally impedes heat loss (Katic et al., 2017). ANs lose vast amounts of body fat (~71.3% compared to HC) in the process of their weight loss (Onur et al., 2005). This reduction in insulating fat (Mecklenburg et al., 1974; Davies et al., 1979; Chudecka and Lubkowska, 2016) contributes to higher skin temperatures and increased heat loss, reported in ANs (Chudecka and Lubkowska, 2016).

### Reduced Production of Body Heat: Lower Basal Metabolic Rate

Basal Metabolic Rate (BMR) or resting metabolic rate (RMR) represents the sum of all metabolic processes that occur *during rest* (Silva, 2006). BMR represents the energy cost of sustaining all essential vital functions, such as breathing, heart rate, nerve impulses transmission, and the like (Clapham, 2012). While providing energy to accomplish these life-sustaining functions, large amounts of heat are released into the blood. This heat is used to maintain a constant T_core_. It is estimated that *~50% of daily BMR is expended in maintaining a constant T_core_* (Landsberg, 2012).

Most researchers concur that BMR is significantly lower in ANs compared to HCs. The decrease in BMR has been reported to be approximately 21% (Polito et al., 2000; Onur et al., 2005) to 25% (Casper et al., 1991), to as high as 32% (Bossu et al., 2007).

A reduced BMR is expected due to the weight reduction since less energy is needed to sustain a smaller body. However, this lowering of BMR is *beyond the reductions one would anticipate with weight loss* (Casper et al., 1991). The depressed metabolic rate typically noted in malnourished individuals is related to the loss of metabolically active tissue (~65%), as well as to a decrease in the metabolic rate of the remaining active tissue (~35%; Taylor and Keys, 1950; Landsberg, 2012).

The metabolic reduction in the remaining active tissue is most likely related to the previously mentioned reduced NE levels (Pirke, 1996); NE is an important factor in driving metabolism (Young and Landsberg, 1977b; Landsberg, 2006). Additionally, thyroid hormone plays a crucial role in the control of BMR (Silva, 2006). A reduction in the conversion from T4 to the active L-tri-iodothyronine (T3) is also a significant determinant of BMR (Silva, 1995; Onur et al., 2005). Onur et al. (2005) reported a 33.4% decrease in T3 in ANs compared to HCs; others concur (Vigersky et al., 1976; Silva, 2006; Misra and Klibanski, 2014).

Of interest is the strong relationship between BMR and T_core_, such as occurs during fever (Landsberg et al., 2009). That is, each degree centigrade rise in temperature is associated with a 10–13% increment in BMR (Landsberg et al., 2009). Similarly, a fall in T_core_ is associated with a reduction in metabolic rate.

A further demonstration of the close relationship between T_core_ and metabolism is evident during “daily torpor,” a condition noted in certain mammals such as hamsters (“torpor” is a state of decreased physiological activity and represents a daily “rest period”). Daily torpor is often associated with a ~20% reduction in BMR and a 0.5°C–2°C decrease in T_core_ (Heldmaier et al., 2004). These values are remarkably similar to those reported in ANs. “The transition into hypometabolism is initiated by *a rapid depression of metabolic rate which clearly precedes the development of hypothermia*” (Heldmaier et al., 2004). Daily torpor is a strategy that enables animals in the wild to survive periods of reduced food availability. It is proposed that the reduction in BMR and T_core_ seen in AN does not represent daily torpor *per se* but is a regulated physiological response (Landsberg et al., 2009) that assists the malnourished individual in preserving scarce body food stores.

In summary: it is reasoned that the primary factor driving the reduction in T_core_ is the reduction in BMR. This is exacerbated by heat lost from the body due to reduced vasoconstriction and reduced insulation.

If metabolic heat is insufficient to support T_core_ in ANs, *what alternate strategies are available* to supplement this shortfall? The following discussion will focus on possible physiological strategies available to HCs, and questions whether ANs can utilize such strategies to thermoregulate.

## Alternate Strategies to Produce and Retain Metabolic Heat

If HCs become hypothermic, the SNS may activate brown adipose tissue (BAT). This highly effective “strategy” results in non-shivering thermogenesis (NST), which produces large amounts of metabolic heat (Schlader and Vargas, 2019). However, when ANs, were compared with HCs controls (Bredella et al., 2012), most ANs did not have cold-activated BAT. Therefore, activation of BAT is not a strategy available to most ANs for generating heat.

In HCs, if NST is insufficient to maintain T_core_ of ~37°C, shivering thermogenesis (ST) may occur. ST involves rapid, involuntary, oscillating muscles, which results in heat production (Schlader and Vargas, 2019) and may produce heat equivalent to 4–5 times the BMR (Tansey and Johnson, 2015). However, in response to acute hypothermia, no ANs displayed observable ST (Vigersky et al., 1976), even in the presence of a low and falling T_core_. Others agree that this does not appear to be a physiological strategy used by ANs, even after substantial body weight gains (Mecklenburg et al., 1974).

Piloerection (goosebumps) is an additional thermoregulatory strategy available to HCs. Piloerection helps retain body heat and may occur in response to increased sympathetic nerve discharge, causing the arrector pili muscles at the base of tiny hairs in the skin to contract, resulting in the hair becoming upright. Upright hairs trap air and thus provide an insulating layer of warm air around the body, minimizing heat loss. However, as humans possess relatively little body hair and are usually clothed, heat conservation through piloerection is generally regarded as insignificant (Schlader and Vargas, 2019).

Lanugo body hair, a characteristic unique to most ANs, presents as long, fine, downy, pigmented hairs on the back of the trunk, the abdomen, and the forearms (Lampert and Lau, 1976; Strumia, 2013). It may assist with thermoregulation by retention of body heat. Schulze et al. (1999) reported diffuse lanugo in 70% of their ANs. They suggested that lanugo’s advantage to the ANs could enhance piloerection by enabling a larger surface area to trap air and help insulate the body. Also, in the absence of piloerection, it is proposed that lanugo may assist in maintaining body heat by acting as a “warm-coat-insulator,” as is the case with many animals. However, this too is not a highly effective strategy for the retention of body heat.

Based on the above discussion, NST, ST, piloerection, and lanugo strategies are limited in assisting ANs with maintaining T_core_. What alternative strategies are accessible to increase metabolic heat to support the maintenance of T_core_ in ANs? It is well established that *physical movement can* increase internal metabolic heat production by 10–20 times, compared to resting values (Saltin and Hermansen, 1966). So physical movement could contribute to the maintenance of T_core_ in ANs (Garland et al., 2011). Several researchers have previously proposed that ANs engage in HyAc to generate metabolic heat (Gutierrez and Vazquez, 2001; Gutierrez et al., 2002; Carrera and Gutierrez, 2018). This thermoregulatory strategy falls under the rubric of *Behavioral Thermoregulation*.

## Behavioral Thermoregulation and Hyperactivity

Thermo-behavior is by far the most frequently used and most effective approach employed by *all humans* to thermoregulate (Romanovsky, 2007; Schlader et al., 2009; Flouris, 2011; Schlader, 2014; Schlader and Vargas, 2019). This strategy includes *all* behaviors that contribute to increasing (or reducing) the heat load. It may include “simple” actions such as putting on or taking off clothing, moving from shady to sunlit areas, curling up or stretching out, or drinking a cup of hot or iced water. It may also include more “complex” actions such as donning a spacesuit, building shelters, and using heating and cooling systems (Schlader and Vargas, 2019). *Thermo-behavior also includes* *adjusting levels of physical activity*. Biologists label the strategy of using movement to provide heat for T_core_ as “activity-thermoregulatory heat substitution” (Humphries and Careau, 2011). Endotherms, including humans, frequently generate metabolic heat *through activity*, which can substitute for a deficit in internal heat necessary to maintain T_core_.

With increased movement, there is an increased need to synthesize and utilize the energy-rich molecule adenosine triphosphate (ATP). Increased utilization of ATP increases metabolic rate. Although large portions of increased metabolic rate provide chemical energy to support movement *per se (~40%)*, a substantial amount of this energy (~60%) is transformed into heat energy. This heat energy is transferred to the blood and assists with maintenance and/or increase of body core temperature in mammals (Landsberg, 2012).

It is proposed that most hypothermic ANs use HyAc as the *primary* behavioral thermoregulatory strategy to assist with maintaining T_core_. The amount of metabolic heat generated would depend on the amount of muscle involved and the intensity and duration of the contractions.

### ANs and Hyperactivity

Over the last few decades, there have been numerous accounts of ANs performing extreme amounts of exercise (Casper et al., 1991; Davis et al., 1994; Gutierrez et al., 2002; Casper, 2006, 2016; Dittmer et al., 2018; Rizk et al., 2020). Many assume that this intense involvement in exercise is a recent phenomenon. However, this unusual display of HyAc has been noted for several centuries (Casper, 2006). Over 400 years ago, many individuals, usually females, considered fasting an act of extreme piety. An extreme amount of physical activity was frequently associated with this saintly self-starvation (Bell, 1985). One description is as follows: “She drank only a little cold water and chewed on bitter herbs yet until the very end, at the opportunity to honor God or do an act of charity, she became robust, vigorously outwalked her companions, and never grew tired—in short, she became hyperactive.” During this period, religious fervor was considered the drive for HyAc (Casper, 2018). Presently many believe that HyAc is motivated predominantly by the desire for weight loss. However, Beumont et al. (1987) suggest that beliefs current at the time strongly influence the analysis of behaviors. Most likely contemporary fitness culture and fashionable thinness may reflect the “flavor of the day” and could influence the responses of ANs *and* influence interpretation by researchers (Kron et al., 1978).

Gull (1997) was the first to report on HyAc in the medical literature. He described a patient who “complained of no pain but was restless and active … it hardly seemed possible that a body so wasted could undergo the exercise which seemed so agreeable.” Gull (1997) further noted that this “peculiar restlessness was difficult to control despite extreme emaciation and weakness.”

Crisp (1967) described similar behavior. He reported that ANs often “walked great distances,” displayed “intense inner restlessness,” spent time doing “vigorous physical exercises,” and could “be heard by their parents pacing up and down in their bedrooms at night. Kron et al. (1978) reported on patients who described themselves as “literally unable to sit still …unable to read or watch television for even a few minute” they felt compelled to pace or exercise. Davis et al. (1994) had patients describe their need to be physically active as “beyond my control” and “nothing would prevent me from exercising”; most would spend many hours a day walking, jogging, or cycling. Rizk et al. (2020) summarized 47 studies published between 1985–2018 and concluded that most ANs demonstrated abnormally high physical activity levels.

This increased urge for movement remained present even after significant weight loss. Furthermore, it only diminished when BMI dropped below 12.5 (Casper et al., 2020). “It is unusual to find a severely emaciated patient (BMI ≤ 15 or below) who is not overactive” (Beumont et al., 1994). A typical pattern is that the activity level increases as weight decreases (Davis et al., 1994) until the patient becomes too weak to perform any but the most basic tasks (Beumont et al., 1994). There appears to be an association between the degree of food restriction and recorded physical activity and restlessness in AN; physical activity and starvation seem to potentiate each other (Davis et al., 1994; Thorburn and Proietto, 2000; Casper et al., 2020). Furthermore, many ANs continue to display driven and rigid exercise behaviors despite detrimental effects on general functioning (Casper, 2020).

This HyAc is puzzling, especially when compared to the behavior of emaciated semi-starved individuals. Semi-starved individuals typically move slowly, are lethargic, tire quickly, and generally reduce movements to a minimum; that is, they engage in energy-conserving behaviors (Taylor and Keys, 1950; Casper and Davis, 1977; Belak et al., 2017). An additional contrast with semi-starved individuals is the mood state of many ANs. ANs frequently report feeling vigorous, seem to have an inexhaustible reservoir of energy, and often pursue various activities with an almost “driven obsessive quality” (Casper and Davis, 1977; Casper, 2020).

Although the Diagnostic and Statistical Manual-V, published in 2013, does not include hyperactivity as a primary symptom (Gummer et al., 2015), HyAc is hugely consequential. It is one of the first signs to appear and the last to subside (Kron et al., 1978). Approximately 1 year before the detection of AN, there is a large increase in the amount of PA (Rizk et al., 2020). On entry into a program, the prognosis is worse if the AN is engaged in HyAc (Dalle Grave, 2009), the hospital stay is more extended, and the drop-out rate increases (Rizk et al., 2020). On discharge, hyperactive ANs are at a higher risk for relapse, and there is a shorter time to relapse (Nagata et al., 2017). A high level of activity in young children appears to be a risk factor for AN (Davis, 1997; Meyer et al., 2008). The level of physical activity also appears to have a biological/genetic component (Lightfoot et al., 2018).

Kostrzewa et al. (2013) reported that the odds of ever being diagnosed with an eating disorder are 2.5 times greater for ANs who displayed HyAc compared to individuals with lower activity levels. They suggested that HyAc may be an important risk factor for developing eating disorders in women. Furthermore, disordered eating is more prevalent among athletes than non-athletes, with 6–45% of female athletes diagnosed with an eating disorder (Bratland-Sanda and Sundgot-Borgen, 2013; Joy et al., 2016). Female athletes are 10 times more likely than non-athletes to be diagnosed with an eating disorder (Bergh et al., 2013). A possible explanation is that involvement in high exercise levels “allows” those with a genetic vulnerability to express this AN behavior. Cook and Hausenblas (2008) suggest a close interaction between exercise dependence and eating pathology; others support this interrelatedness (Davis et al., 1994; Thorburn and Proietto, 2000; Casper et al., 2020).

### Proposed Classification of Hyperactivity

There are several concerns associated with hyperactivity research (Cook and Hausenblas, 2008; Meyer et al., 2011; Rizk et al., 2020). At present, there is no consensus regarding which terms best describe this phenomenon (Kron et al., 1978; Beumont et al., 1994; Davis et al., 1994; Dittmer et al., 2018; Rizk et al., 2020). It is variously referred to as “hyperactivity,” “compulsive exercise,” “over-exercise,” “unhealthy exercise,” “drive to exercise,” “motor restlessness,” “exercise dependence,” “high-level physical activity,” “diffuse restlessness,” “paradoxical liveliness,” and “abundance of physical energy.”

In an attempt to standardize terminology, it is proposed that the term *hyperactivity* (HyAc) includes all activities displayed by ANs. HyAc may then be *subdivided* into three overlapping categories that span low-intensity movements to high-intensity exercise. These categories are Excessive Exercise (ExEx), Non-Exercise Thermogenic Activity (NEAT), and Spontaneous Physical Activity (SPA). The following definitions differ somewhat from those presented by Levine and Kotz (2005) and Teske et al. (2008) and are more in line with the three subtypes presented by Dittmer et al. (2018).

#### Excessive Exercise (ExEx)

Includes activities that are deliberate, conscious, and goal-directed (Davis et al., 1994), at least initially (Rizk et al., 2020). However, over time, exercise may not be under voluntary cognitive control but may represent a “subconscious biological drive,” with this activity “becoming totally automatic” (Dittmer et al., 2018; Rizk et al., 2020).

Activities may include gymnastics, ice skating, jogging/running, cycling, calisthenics, yoga, dance (especially ballet), and weight training, to name a few. Participation may include team sports such as volleyball, basketball, and soccer. Activities may also include *low-intensity, long-duration exercises*, such as prolonged walking or cycling. Involvement may be daily for several hours per day (Crisp, 1967; Casper and Davis, 1977; Kron et al., 1978; Dittmer et al., 2018; Rizk et al., 2020). The amount of metabolic heat produced would depend on the amount of muscle involved and the intensity and duration of the activity.

#### Non-exercise Activity Thermogenesis (NEAT)

In ANs (*and HCs*), NEAT includes all activities of daily living *not classified* as regular exercise/ExEx (Levine, 2007; Gummer et al., 2015). NEAT could include activities such as dressing, bathing, house-cleaning, washing dishes, cooking, working at a computer, driving a car, walking to the mailbox, and the like (Beumont et al., 1994; Chieffi et al., 2017; Dittmer et al., 2018). It would also include leisure time-recreational activities such as 10-pin bowling and occupational activities (Lightfoot et al., 2018).

**Figure 1:**
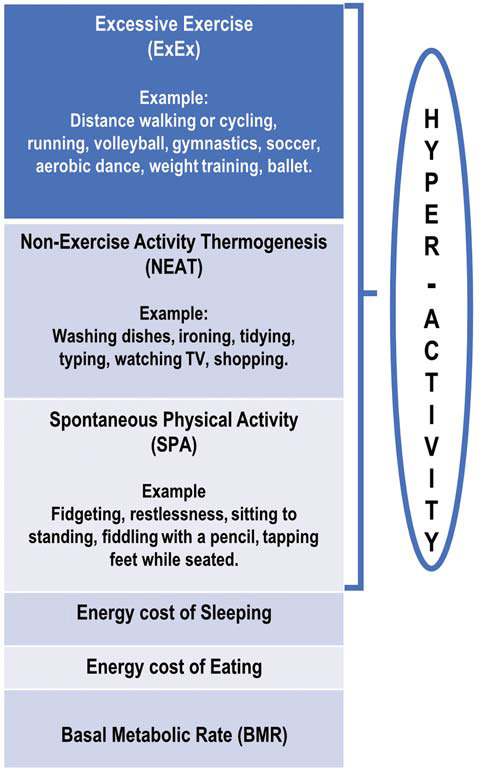
Schematic diagram of proposed divisions of daily energy expenditure in anorexia nervosa patients.

In ANs, NEAT may be excessive and may include unnecessary energy expenditure (Figure 1) such as taking the stairs instead of an elevator, biking to school instead of taking a bus, engaging in strenuous housework and yard work, and pacing while watching TV (Casper, 2018). ANs appear to expend considerable energy in this NEAT category (Beumont et al., 1994; Gummer et al., 2015), although no research has specifically assessed this.

#### Spontaneous Physical Activity (SPA)

In ANs, SPA is defined as movement that is not goal-oriented but rather is an expression of an inherent drive for activity. It includes a display of “restless” behaviors while engaging in everyday activities. It consists of activities such as gesturing, shifting in one’s seat/dynamic sitting (van der Berg et al., 2019), frequently moving from sit-to-stand, fidgeting while sitting or standing, swinging legs or tapping feet while seated, fiddling with a pencil, rubbing hands together, picking at nails, deliberately tensing abdominal or leg muscles, and also gum chewing (Crisp, 1967; Kron et al., 1978; Gull, 1997; Belak et al., 2017; Kotz et al., 2017; Dittmer et al., 2018; Casper et al., 2020). These SPA activities would also generate metabolic heat, probably to a lesser degree compared to NEAT (van der Berg et al., 2019).

It is proposed that SPA be defined as movements that *increase energy expenditure with* *no change in the center of gravity (CoG) in a horizontal plane*. For example, moving from sitting to standing will incur a *vertical* displacement of CoG and be classified as SPA. If an individual is sitting or standing and simultaneously fidgeting, this will incur additional energy expenditure and could be classified as SPA (Levine et al., 2000).

To more objectively assess this category of SPA, Belak et al. (2017) used a shoe-based-accelerometer to compare foot-fidgeting behavior in ANs relative to HCs, while eating, while filling out questionnaires, and while watching television. They reported that ANs engaged in significantly more fidgeting behavior than HCs during all three tasks.

Possibly, ANs who do not engage in ExEx expend substantial energy performing NEAT and SPA (Garland et al., 2011). This has not been assessed.

This distinction between different HyAc categories (ExEx/NEAT/SPA) is not always clear-cut (Garland et al., 2011). Theoretically and practically, there is extensive overlap between ExEx, NEAT, and SPA. For example, if an individual walks 4 km per day to and from work/school, should this be classified as ExEx or NEAT? If an individual remains standing while watching TV, should this be classified as NEAT or SPA? The reader is referred to an excellent discussion on these subdivisions of hyperactivity by Dittmer et al. (2018).

In summary, anecdotal and research-related accounts agree that *a vast majority of ANs engage in ExEx, NEAT, and SPA to varying degrees*. These activities generate metabolic heat to varying degrees, which is proportionate to the amount of muscle movement.

Although it has been hypothesized that ExEx is the primary thermo-behavior used by ANs, a crucial question is *what initiates ExEx*? What “compels” the AN to begin and to continue exercising? It will now be proposed that the sensation of “cold hands” is central to the repeated initiation of ExEx (A later section will address the *stimulus* for NEAT and SPA).

## Role of “Cold Hands” in Activation of ExEx

### ANs Have Cold Hands

Besides having a T_core_ significantly lower than HCs, cold hands and feet and peripheral cyanoses are common physical features of AN, even in a thermally neutral environment (Davies et al., 1979). This observation has been confirmed by many (Wakeling and Russell, 1970; Lampert and Lau, 1976; Freyschuss et al., 1978; Luck and Wakeling, 1980, 1981; Crisp, 1984; Gull, 1997; Strumia, 2013; Bergersen and Walloe, 2018).

Chudecka and Lubkowska (2016), who assessed skin temperature over most areas of the body, demonstrated that *skin temperatures for the front and back of the hands were significantly lower for ANs* compared to HCs (approximately 2°C lower). These were the *only body areas reported to be lower for ANs compared to HCs!* All other body surfaces were either significantly *higher* or *not* significantly different for ANs compared to HCs.

This sensation of cold hands is most likely related to the condition of acrocyanosis (Das and Maiti, 2013), frequently diagnosed in ANs by dermatologists (Strumia, 2013) and which may be present in as many as 81% of female patients, with a female to male ratio of 6:1.

Acrocyanosis is a disorder of the peripheral circulation, characterized by blueness and *coldness*, mainly of the hands and feet (Bergersen and Walloe, 2018). It usually presents as a persistent, symmetric, painless discoloration (Bergersen and Walloe, 2018). There is no loss of function, and it is mainly considered a benign cosmetic problem. It usually resolves spontaneously when the underlying cause is eliminated; in the case of ANs, this would be weight gain. No standard treatment is available, but it is suggested that hands should be kept warm (Das and Maiti, 2013). In addition to acrocyanosis, Raynaud’s phenomenon has also been reported in ANs; in this condition, fingers feel cold and numb and are initially white, followed by turning blue (Bergersen and Walloe, 2018).

### Cold Hands Induce the Overall Sensation of “Chilliness”

Besides having “cold hands,” ANs frequently complain of experiencing body “chilliness.” Swenne and Engstrom (2005) reported that on entry into a treatment program, the *most common* symptom, acknowledged by ANs, was the sensation of “chilliness” (84% of 211 subjects), while the most common sign, noted by investigators, was “peripheral hypothermia” (80% of 211 subjects). Furthermore, they reported a significant relationship between reports of “chilliness” and observations of “peripheral hypothermia” and “peripheral cyanosis” (blue extremities).

It is now suggested that in ANs, there is a relationship between cold hands and the overall internal experience of “chilliness”; that is, the sensation of cold hands drives the overall body sensation of chilliness and *not* the low T_core_.

The idea that a *discrete body area* such as “cold hands” could determine the overall perception of temperature is supported in part by Mundel et al. (2016). They reported that when HCs (males) exercised in the cold, head-surface-temperature modulated thermal behavior/exercise intensity. Subjects subconsciously “monitored” head surface temperature and adjusted their exercise intensity based on this perception. They concluded that *peripheral skin temperature* regulates thermal behavior in the cold.

### The Hand Design Is Optimal for Assisting With Thermoregulation

The hands’ anatomical and physiological features are exquisitely designed to support heat conservation (or dissipation; Taylor et al., 2014). In general, the higher the ratio of surface-to-mass, the more effectively heat can be conserved (and dissipated). The surface-area-to-mass ratio of each hand is 4–5 times greater than other areas of the body (Taylor et al., 2014; Walloe, 2015).

An additional adaptation of the hands is related to the skin covering, which is specialized for thermoregulating (Bergersen and Walloe, 2018). The skin surfaces may be divided into glabrous (non-hairy/palm) and non-glabrous (hairy back) skin (Romanovsky, 2014; Filingeri, 2016). Glabrous skin includes the palms of the hands and fingers and soles of the feet and toes (Romanovsky, 2014).

Glabrous skin contains specialized structures, arteriovenous anastomoses (AVAs). AVAs form direct links between arterioles and venules with no capillary section (Lossius et al., 1993; Walloe, 2015). They are typically 10 times the size of capillaries (Walloe, 2015) and may convey about 10,000 times as much blood as a comparable length of the capillary (Lossius et al., 1993; Walloe, 2015). They function as sphincters and may transition from being completely open to completely closed in response to a change of a few 10ths of a degree centigrade (Bergersen et al., 1997). When temperatures rise sufficiently, AVAs open, and blood flow increases dramatically (Walloe, 2015).

In human glabrous skin, the largest AVAs are in the palms and the soles (Bergersen and Walloe, 2018). These structures play a role in temperature regulation (Walloe, 2015) and regulation of vascular pressure (Lossius et al., 1993).

In HCs, when exposed to low ambient temperature, the SNS activates a rhythmic opening and closing of AVAs; this is a defensive response that aims to preserve blood flow to the extremities and prevents tissue injury during cold exposure (Lossius et al., 1993). *Presumably*, in non-exercising hypothermic ANs, with reduced ability to activate the SNS and low blood pressure/blood flow, these normal physiologic fluctuations would not occur; thus, AVAs would remain mainly closed, resulting in reduced blood flow (Bergersen and Walloe, 2018). “During hypothermia, the extremities may be physiologically “isolated” to restrict heat loss…” (Taylor et al., 2014). This condition may be referred to as “acral” coldness, which describes a condition wherein fingers (or toes) are exceptionally cold (Bergersen and Walloe, 2018).

One can speculate that in ANs, in response to ExEx, there is an increase in overall metabolic rate and blood pressure, resulting in the movement of warmer (Funk et al., 1994) blood from the core to the periphery/hands (Stromme et al., 1963). Increased perfusion (Stoner et al., 1991) could assist with the passive opening of AVAs (vasodilation). The release of nitric oxide, a locally synthesized vasodilator from cells in the AVAs walls, may also mediate dilation (Funk et al., 1994). Increased perfusion of hands with warm blood would increase local hand temperature (Stoner et al., 1991), a “pleasurable” outcome (Cabanac, 1971).

Although hands play a vital role in the whole-body thermoregulatory response to cold (Flouris et al., 2006), the relationship between hand temperature and T_core_ is unclear (Brajkovic et al., 1998). Caldwell et al. (2014) suggest that it may be challenging to restore blood flow to hands when T_core_ is extremely low, a condition known as “dilation resistance.” Much research is needed to determine ANs’ hand temperature response during exercise and in different ambient temperatures (Davies et al., 1979).

### Role of Skin Temperature in Thermal Perception

Although T_core_ is maintained within a narrow temperature range, this does not apply to the external skin temperatures. HCs can maintain a T_core_ of ~37°C over a range of skin temperatures that may vary between 20°C–40°C and ambient temperatures between 15°C and 54°C (Romanovsky, 2014; Filingeri, 2016).

The benefit of this is that changes in T_skin_ are more responsive to fluctuations in ambient temperatures, unlike T_core,_ which changes less rapidly (Weiss and Laties, 1961). Thus, the T_skin_ acts as a rapid “warning signal” for the central nervous system, alerting it to possible fluctuations in ambient temperatures that could adversely impact T_core_ (Schlader et al., 2011, 2013).

It is now widely accepted that *skin temperature* (Romanovsky, 2007; Flouris, 2011) is the primary input for the *initiation of thermo-behavior* (Schlader et al., 2009, 2011, 2013; Schlader and Vargas, 2019). It is now suggested that *in ANs, instead of ambient temperatures*, the input from “*cold hands*” initiates thermo-behavior. This thermal input would occur *via* activation of specialized cold-receptors in the skin.

### Activation of TRPM8 Thermo-Receptors

A specific population of neurons expresses Transient Receptor Potential, subfamily Melastatin (TRPM8) channels (Filingeri, 2016). These neurons terminate in or immediately beneath the epidermis in the skin (Schlader and Vargas, 2019). Activation of these receptors allows afferent nerves to transduce temperature stimuli into action potentials (Filingeri, 2016). Non-noxious (non-painful), moderate cold stimuli activate TRPM8 channels. Various centers in the CNS receive these nerve impulses (Schlader and Vargas, 2019). “It is generally believed that changes in thermal sensation are dictated by skin temperature, independent of core temperature” (Schlader and Vargas, 2019).

However, human temperature sensing is not homogenous across the body but differs significantly depending on the skin regions. Filingeri et al. (2018) used HCs (females and males) to compare cold sensitivity over glabrous (palms) and non-glabrous (hairy back) skin of hands and feet. Hands were twice as thermosensitive for cold as feet in both genders, while non-glabrous skin was more cold-sensitive than glabrous skin. If these results apply to female ANs, it suggests that AVAs of glabrous skin reduce blood flow to the hands resulting in acral cold, while hand hairy skin is the primary detector of coldness in the hands.

### Central Processing of Cold Sensations

The brain areas responsible for integrating peripheral/skin thermal signals and converting them into behavioral responses have not been elucidated (Almeida et al., 2015). However, recently, Schlader and Vargas (2019) have proposed that the initiation of thermo-behavior involves several input signals. First, an individual must register a “thermal *sensation*” (for ANs this would be cold hands). This signal is “interpreted” subconsciously as “thermal *discomfort*.” The affective (emotional) sensation of “discomfort” *motivates* the “*decision to behave*,” and “*thermo-behavior*” *is initiated* (in ANs this would be HyAc). The transformation of the initial innocuous sensation of “cold” to a “warm/comfortable” sensation is a pleasurable/rewarding outcome (Cabanac, 1971).

In summary, the closure of AVAs reduces local hand temperature in ANs. TRPM8 receptors then *provide the first source of thermal awareness* and activate heat-seeking behavior. The “aim” of this behavior is to increase hand temperature and, more importantly, although most likely not a conscious perception, preserve T_core_.

## CNS Alterations Associated With an and HyAc

It has previously been suggested that “cold hands” are the *primary stimulus* for initiating ExEx. It is now proposed that *additional factors* promote and sustain ExEx as well as NEAT and SPA. *These factors involve the synthesis of specific brain neuromodulators*, which create a “unique” brain profile in genetically-prone ANs (Levine and Kotz, 2005; Teske et al., 2008). In addition to impacting various aspects of HyAc, the actions of these central neuromodulators may *explain other unusual mood/behaviors seen in ANs*.

The two neuromodulators that will receive *cursory* attention are corticotrophin-releasing hormone (CRH) and the orexin system (orexin-A). Undoubtedly many other factors are involved.

### The Proposed Role of Central Corticotropin-Releasing Hormone in AN

CRH is typically related to the stress response and activation of the HPA axis, resulting in elevated glucocorticoids in the systemic circulation (Hotta et al., 1986). Besides having many peripheral effects, CRH has extensive *central effects* that impact mood and behavior (Dedic et al., 2018). In humans, central levels of CRH are assessed *via* measurement in CSF. ANs have significant elevations of CRF levels in CSF compared to HCs (Hotta et al., 1986; Kaye et al., 1987).

Typically, rodent models have been used to investigate the effects of brain-CRH on behavior. In rats, wheel-running *increases* *brain levels* *of CRH* (Rivest and Richard, 1990; Dedic et al., 2018). Conversely, elevated brain levels of CRH *increase running activity* (Sutton et al., 1982; Kawaguchi et al., 2005). If the same running-response applies to ANs as it does to rodents, then elevated, *central CRH* *may partly be responsible for high levels of ExEx seen in ANs* (Hotta et al., 1986; Kaye et al., 1987, 1989; Casper, 2006, 2018; Kaye, 2008).

Rodent wheel-running studies have also demonstrated that elevated central CRH induces a significant *loss of appetite* (Rivest and Richard, 1990; Heinrichs and Richard, 1999; Dedic et al., 2018). If a similar response applies to ANs, this would explain, in part, the apparent loss of appetite displayed by ANs (Misra and Klibanski, 2014). It would also justify the claim of many ANs, who report that exercise “dulls the pain of hunger” (Casper, 2006).

Since the early 20th century, the mammalian CRH family has been extended to include Urocortin (Ucn) I, II, and III, as well as CRH receptor 1 (CRH-R1) and 2 (CRH-R2; Reul and Holsboer, 2002; Bale and Vale, 2004). The classic central stress response involves the binding of CRH-CRH-R1, which appears to induce anxiety-like behaviors. However, the central binding of Urc II and III with CRH-R2 in discrete areas of the rat brain induces an *anxiolytic effect* (Casper, 2006). That is, CRH-R2 appears to regulate stress-coping behavior (Bale and Vale, 2004) that *opposes* the general anxiogenic effect of CRH-CRH-R1 (Kishimoto et al., 2000; Kormos and Gaszner, 2013; Scharner et al., 2018). If this pertains to ANs (Kaye, 2008), that is, Urc II and III activate CRH-R2, it will give credence to the frequently observed yet inexplicable “lack of concern,” “liveliness,” “sense of contentment,” and “denial of the seriousness of their condition” displayed by ANs. This attitude is perplexing, considering their dangerously low body weight and associated health-related conditions (Casper, 2006; Scheurink et al., 2010).

In summary, it is proposed that HyAc increases central levels of CRH, which in turn stimulates HyAc. In addition, elevated central CRH may be involved in appetite suppression and the “unusual” anxiolytic responses seen in ANs.

### Orexins, NEAT, and SPA

There is significant evidence that the CRF system and restricted feeding in rats (Kurose et al., 2002), activates the orexin (hypocretin) system (Winsky-Sommerer et al., 2004). While CRF and related peptides are found in discrete areas of the CNS, orexin neurons and receptors are widely dispersed throughout the CNS, resulting in multiple physiological processes.

Of particular interest is orexin-A, which plays a crucial role in *arousal and alertness* (Sargin, 2019). Arousal and alertness may be the underpinnings of restless/fidgeting behavior associated with SPA and NEAT (Scheurink et al., 2010; Kotz et al., 2017). Teske and Mavanji (2012) and Kotz et al. (2017) have demonstrated in extensive rodent studies that the orexin-A is “irrefutably” responsible for heightened arousal/vigilance. Kiwaki et al. (2004) reported that injecting orexin-A into rat brain was associated with a dramatic increase in SPA and NEAT behavior; this occurred in a dose-dependent fashion. Others support the concept that orexin-A stimulates SPA and energy expenditure (Teske and Mavanji, 2012).

Related to the concept of a heightened state of arousal is the hypothesis that AN represents an ancient behavioral strategy evoked during food shortages (Guisinger, 2003; Barson, 2020). This state would encourage genetically prone individuals to leave depleted food areas in search of new “food patches” (Boer et al., 1990; Guisinger, 2003). Heightened arousal/vigilance would also encourage “foraging” behavior, a functional asset (Barson, 2020).

There is also a robust connection between sleep and orexin-A. Reduced synthesis of orexin-A induces the pathological condition of narcolepsy, a chronic sleep disorder that causes excessive daytime drowsiness (Sakurai, 2003). On the other hand, increased levels of orexin-A induce wakefulness (Sakurai, 2002). Crisp (1984) investigated sleep patterns in ANs and reported that ANs display light, restless sleep with early morning wakings. Sauchelli et al. (2016) reported an association between plasma concentrations of orexin-A and greater sleep disturbances in ANs.

It has recently been proposed that activation of the orexin system promotes anxiolytic and anti-depressant behavior in rats (Summers et al., 2020). This would support the anxiolytic, anti-depressant effect associated with CRH-R2 (Kishimoto et al., 2000; Kormos and Gaszner, 2013; Scharner et al., 2018).

In addition to the above actions, the orexin system may be involved in the brain reward circuitry *via* interaction with the dopaminergic reward system (Scheurink et al., 2010). Reward center activation has been implicated in AN (Sodersten et al., 2016).

However, the few studies that have examined plasma orexin-A levels in ANs have yielded mixed findings. Bronsky et al. (2011) demonstrated that orexin-A levels were higher in adolescence with AN than HC. Sauchelli et al. (2016) found no differences in plasma orexin-A levels in AN compared to HC. However, plasma orexin levels do appear to decrease during 3- to 6- months treatment (Janas-Kozik et al., 2011) and during 8 weeks of refeeding (Bronsky et al., 2011). Thus limited evidence suggests that orexin-A levels may be altered in AN and normalize as patients recover. Additional research is needed in this area (Berner et al., 2019).

In summary, ExEx increases central levels of CRH. Increased CRH is involved in the activation of the orexin system. Elevated orexin-A levels in ANs may account for the high levels of restless behavior, manifesting as SPA and NEAT. In addition, elevated orexin-A in ANs may explain sleep disturbances, anti-depressant behavior, and activation of reward centers.

## Thermoregulatory Behavior: A Formed Habit

The execution of thermoregulatory behavior in mammals may be classified as “motivated” behavior. That is, the outcome of thermo-behavior can serve as a “reward” to motivate the execution of “new” thermo-behaviors (Tan and Knight, 2018). For example, if a rat is exposed to cold (*stimulus*), it can learn to lever-press to turn on a heat lamp (*response*); the generation of heat is then “rewarding” (*outcome*) to the cold rat (Weiss and Laties, 1961). Similarly, if a rat is exposed to heat, it can learn to lever-press to turn on a “rewarding” cold shower (Epstein and Milestone, 1968). Thus, by utilizing “rewards,” new behaviors/habits may be formed.

**Figure 2:**
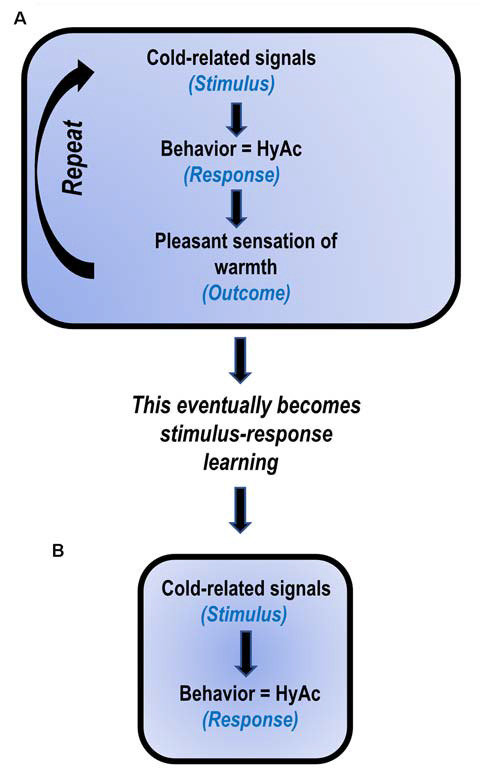
Proposed model: HyperActivity (HyAc) a developed habit (based on Uniacke et al., 2018). **(A)** In the early stages, HyAc involves *stimulus-response-outcome* learning. **(B)** After daily repetition for extended periods, the behavior becomes less sensitive to the *outcome* and only involves *stimulus-response* learning. It then automatically occurs when exposed to cold-related cues.

Uniacke et al. (2018) have suggested that *habit formation* may play a key role in the intransigence of AN (Figure 2). The present author suggests that *habit formation* is central to the *development and maintenance of HyAc*. That is, cold-related signals (*stimulus*) result in the initiation of HyAc-behavior (*response*). Engagement in HyAc generates pleasant/rewarding body-heat (*outcome*).

Continued repetition of these events subsequently results in stimulus-response learning (Uniacke et al., 2018). In this instance, the *two events*, cold-related signals *(stimulus*) and HyAc (*response*) become strongly associated, and the previous rewarding *outcome* of “warmth” is not necessary to maintain this behavior. Thus, stimulus-response learning may serve as an explanation for the frequently observed, at times implausible, compulsive drive for HyAc, seen in so many ANs (Casper, 2018; Rizk et al., 2020).

Although it has been suggested that the *initial* motivation for ANs to engage in HyAc may be “cold sensations,” *physical activity per se* may generate numerous *additional* “*rewards*” (Scheurink et al., 2010; Garland et al., 2011). These “rewards” could include stress reduction, increased anxiolysis, and mood improvement. Endurance exercise may also produce a sense of euphoria, which could be addictive, and is referred to as a “runners’ high” (Hicks et al., 2019).

## Research Supporting Heat Treatment In

### Rodent Studies

The most promising results regarding the role of heat in the treatment of hypothermic ANs, come from rodent studies using the activity-based anorexia (ABA) model (Gutierrez et al., 2006, 2009; Carrera et al., 2012; Cerrato et al., 2012). ABA is an animal model of *self-starvation* and *increased wheel running*, similar to what occurs in human ANs who perform ExEx. This rodent model displays many additional AN-disorder signs, including drastic weight-loss, hypothermia, disturbed sleep, and alterations in various neuroendocrine axes, as well as alterations in various appetite-regulating hormones.

Briefly, the ABA model involves placing a rat on a restricted feeding schedule (~1 h per day instead of *ad-lib feeding*) and giving free access to a running wheel. The rat will begin to eat less each day while they continue running, resulting in a dramatic weight loss. If the experiment is not terminated, after about 7 days, ABA-rats will continue to run until they die of starvation. According to Pare (1977), a possible answer to this *an-homeostatic* behavior may be the fact that “body temperature is low in these hungry rats and…running represents an attempt to increase and maintain body temperature at a normal level.”

Hillebrand et al. (2005) conducted a seminal study using the ABA model. Their primary purpose was to examine whether hyperactivity was a thermoregulatory behavior aimed at preventing starvation-induced hypothermia. They proposed that if excessive wheel running is associated with thermoregulation in ABA rats, making heat available to these rats would prevent hyperactivity and starvation-induced hypothermia, and weight loss.

To test this hypothesis, 13 female Wistar rats had thermo-transmitters surgically implanted in the abdominal cavity to monitor T_core_. After recovery, researchers assigned rats to one of two groups. A *non-warm-plate group*, representing a typical ABA set-up, was placed in a cage consisting of a “residual” living area and open access to a running wheel. A *warm-plate group* had the cage area divided into three sections: a residual area, a running wheel area, and 20% of the cage containing a 37°C heating plate (This heat did not impact ambient temperature).

When both groups were exposed to restricted 1-h feeding per day, the outcome was as follows:

- The warm-plate rats retained their *body temperature* over the 6 days. However, the non-warm plate rats showed a significant reduction in T_core,_ staring at 24 h; T_core_ continued to decrease through the 6 days of the experiment.
- Total *running wheel activity* was significantly less for the warm-plate rats compared to the non-warm-plate-group.
- *Bodyweight* was significantly different. The warm-plate rats only lost 18.8%, while the non-warm-plate group lost 24.8% body weight (the latter being similar to what ANs lose).
- *Food intake* was not significantly different between the groups.
- The warm-plate group spent significantly *more time on the warm-plate* during the restricted feeding period.

In summary, access to the warm plate prevented hypothermia, hyperactivity, and body weight loss. The authors concluded that, given the “choice,” rats prefer to *prevent hypothermia passively* by choosing a warm plate rather than actively regulating body temperature by hyperactivity. From these preclinical findings, the authors proposed that heat treatment in hypothermic ANs may be beneficial.

Hillebrand et al. (2005) also questioned whether running predicted a rise in body temperature. They concluded that running was a *good predictor of increased body temperature*. If this applies to ANs, it would support the hypothesis that HyAc aids in maintaining/increasing T_core_.

An additional hypothesis tested in this study was whether a decrease in body temperature occurred *immediately before* the onset of wheel running, demonstrating that a reduction in T_core_ was the biological trigger for hyperactivity. They concluded that there was no evidence to support this hypothesis and that “a decrease in core temperature does not directly stimulate wheel running.” However, an alternate interpretation will now be proposed. If detection of thermal events in the rat is similar to that of humans, “awareness” of temperature changes may be driven by peripheral signals instead of central signals. The rat tail is considered an essential organ for temperature regulation (Gemmell and Hales, 1977; Vanhoutte et al., 2002). It contains hairless-glabrous skin with abundant arterio-venous anastomoses, especially at the base of the tail. Theoretically, the ABA-rat could be “monitoring” changes in skin temperature at the base of the tail (possibly equivalent to the “hand” of the AN), and this would more likely act as a biological trigger for wheel running as opposed to central T_core_. This hypothesis would need to be tested.

Cerrato et al. (2012) reported similar findings using female rats under increasing ambient temperatures (21°C–32°C). They reported that warming ABA rats reversed running activity, maintained food intake, and enabled female rats to recover from acute weight loss. They suggested that these findings “represent strong preclinical evidence in favor of heat supply as a useful adjunctive component for the treatment of AN.” Similar results were reported by Gutierrez (2013).

Additional research, using a rodent model, has suggested that temperature and hyperactivity are closely interconnected (Rixon and Stevenson, 1957; Stevenson and Rixon, 1957; Weiss and Laties, 1961; Gutierrez et al., 2006; Cerrato et al., 2012).

### Human Studies

Presently, there is minimal evidence to support the role of heat treatment in ANs. Gull (1997) claimed to have successfully treated patients by placing “an Indian-rubber tube …filled with hot water along the spine of the patient.” However, he gave no details concerning the length of time this was applied, the frequency, or the water temperature [It is interesting to note that the spinothalamic tract is the first level of central integration of thermo-afferent information within the CNS (Filingeri, 2016)]. Gull (1997) also proposed that patients wear warm clothing and “for a time be kept in a warm bed.”

During the 20th century, there were numerous reports of ANs experiencing a low body temperature and hypothermia, yet few attempts were made to study the impact of heat treatment on hypothermia and hyperactivity. However, in 2002, Gutierrez and Vazquez (2001) successfully treated three *hyperactive* ANs using three different heat treatments. Patient 1 was exposed to a continuously elevated ambient temperature of 25°C for 2 months. Patient 2 wore an electric vest for several hours a day for 6 months; this covered the back *from the neck to the lumbar region*, similar to what Gull (1997) had recommended. Patient 3 used a sauna as a heating device for 5 months; sauna sessions increased from shorter to longer exposures, with increasing temperatures and more frequent weekly sessions. In all three cases, there was an *immediate* *reduction in overactivity*. By the *end of the treatments*, activity had decreased dramatically. When assessed 30 months later, the BMIs for all patients had improved dramatically. That is, BMI increased from <12 to 20 in Patient 1, from 12.3 to 21 in Patient 2, and from 17 to 21.7 in Patient 3. However, these were case reports with no control group; additionally, compliance could not be assessed because the study was conducted on outpatients.

Nevertheless, it was not possible to replicate this result in a subsequent study (Birmingham et al., 2004). In this study, 21 female ANs were randomized to a Heat Group or a Control Group. The heat was only applied for 3 h daily for 21 days, using electrically heated vests around the chest. The outcome did not demonstrate an increase in weight gain with warming. However, in this study, the mean BMI of ANs was 17.7, which may be considered borderline-AN. It is also unclear whether patients were initially deemed hyperactive. These and additional other factors may have confounded the results.

Cerrato et al. (2012) investigated ambient temperature’s role in the amount of daily physical activity performed by ANs. They reported significantly higher levels of activity during the colder months. They concluded that ambient temperature contributes to the expression of excessive physical activity in ANs and suggested that keeping patients warm may moderate the expression of excessive physical activity (Gutierrez and Vazquez, 2001).

Bergh et al. (2013) have developed a treatment program with the primary focus on the normalization of eating behavior instead of the more traditional cognitive interventions. They also incorporated heat treatments, providing ANs with thermal blankets or jackets and allowing them to use warm rooms (temperatures <40°C). Their rationale for doing this was to help calm ANs. However, it is difficult to assess the impact of heat since “heat therapy” only constituted part of their program, and they provided few details regarding this.

Additional factors that may play a role in temperature regulation include latitude (Vazquez et al., 2006), seasonal temperature, and climate (Gutierrez et al., 2013).

At present, the impact of heat treatment as a therapy for hyperactive ANs is minimal. This appears to be a most fruitful area for research.

## Recommendations Based on This Hypothesis

If ANs are HyAc, in an often-unconscious attempt to thermoregulate, and if HyAc is inhibiting recovery, this suggests that removing this “obstacle” would promote recovery. It is therefore proposed that keeping the ANs warm 24/7 would be advantageous. However, it is not proposed that the use of heat should replace traditional psychotherapeutic interventions (Starzomska et al., 2018) or refeeding strategies (Garber et al., 2016). Instead, including “heat-treatment” as an adjuvant strategy may assist recovery (Gutierrez and Vazquez, 2001; Hillebrand et al., 2005; Cerrato et al., 2012).

The following recommendations are based on the premises proposed in this article.

### Increasing the Availability of Heat for the AN Patient

Much emphasis has focused on the role of “cold hands” in initiating thermo-behavior (HyAc), possibly leading the reader to logically deduce that the problem could be resolved by heating the hands. Undoubtedly, the hands need to be kept warm, as has been suggested in the case of acrocyanosis (Nousari et al., 2001). However, cold hands do not represent the entire problem since hands act as “bait” to alert ANs to the need for heat to assist with deep T_core_. So, the primary issue is the need for heat for T_core_. The following strategies are proposed for increasing body heat.

#### To Retain Body Heat

ANs should wear warm clothing, socks, gloves, knitted hats, and such to retain metabolic heat produced in the body. This practice should be adhered to during waking hours and even more so during sleep since T_core_ tends to decrease between 12:00 AM and 6:00 AM (Lacey et al., 1976; Sauchelli et al., 2016).

#### To Increase Absorption of External Heat

Consistently employ whole-body warming by adhering to the following:

#### Indoor

Room temperature may be as high as 32°–35°C [An AN-patient was successfully rehabilitated living at 25°C (Gutierrez and Vazquez, 2001)]. A fruitful area of research would be to determine an *optimal* ambient room temperature.

#### Outdoor Ambient Temperatures

ANs should preferably not spend time outside if temperatures are low unless extremely well clothed. However, when possible, they should be encouraged to sit in the sunshine. This category of “warming” may also include seasonal temperature and geographical latitude (Gutierrez et al., 2002, 2013). If there is an option of relocating to a warmer climate, even temporarily, this is advisable (Gull, 1997; Gutierrez et al., 2002, 2013).

#### Heating-Aids

ANs should be encouraged to use hot-water bottles, heaters, heated vests/gloves/socks/blankets. Take warm showers or baths, use a sauna/hot tub/steam room.

#### Food and Liquids

ANs should ingest warm foods such as soups, warm meals, warm water, and tea.

### To Generate Internal Metabolic Heat

ANs would need to increase muscle activity to increase internal metabolic heat. However, this should be minimized until the AN has regained a healthy weight, and even then, executed with caution (Sauchelli et al., 2015).

### Re-educating Eating Behavior

Most likely, there will be a need to re-educate eating behavior in ANs, as is the case with severely overweight patients (Esfandiari et al., 2018). The experience of AN would undoubtedly have distorted eating patterns, as is the case with obese patients (Sodersten et al., 2015).

### Refeeding and the Thermic Effect of Food on Thermoregulation

Nutritional and weight restoration (refeeding) are core components of many treatment programs for AN (Marzola et al., 2013). Associated with refeeding is the increased energy requirement of TEF (also referred to as Specific Dynamic Action or Dietary Induced Thermogenesis). TEF represents the amount of energy/heat expenditure above the BMR used for food processing and storage. In healthy subjects, with a mixed diet of protein, carbohydrate, and fats, TEF usually represents about 10%–16% of daily energy expenditure (Marzola et al., 2013).

In ANs, especially during the early phases of rehabilitation/refeeding, TEF is significantly higher for ANs than for HCs (Moukaddem et al., 1997) and may represent up to 30% of daily energy expenditure. This elevated level of energy usage is problematic as it requires ANs to ingest significantly more calories to gain weight during rehabilitation (Russell et al., 2001; Marzola et al., 2013).

A variety of reasons have been proposed to account for this increase in TEF, including hormonal and psychological factors (Rigaud et al., 2007), biological repair processes (Russell et al., 2001), and repletion of liver and muscle glycogen stores (Moukaddem et al., 1997).

However, an alternate explanation for elevated TEF may be related to the need for metabolic heat to assist with the restoration of T_core_ in the hypothermic AN. Marzola et al. (2013) suggest there is evidence that “energy intake may be converted into heat, rather than being used to build tissue in AN.” In a study regarding 24 h-circadian body temperature, during nutritional restoration, they reported that ANs became hyperthermic (elevated body temperature) and frequently complained of becoming “hot and sweaty,” particularly during the night when body temperature usually decreases. The researchers speculated that this was most likely a response to high TEF during re-nutrition.

In partial support of this increased availability of heat for T_core_, Moukaddem et al. (1997) found that after approximately 1 week of refeeding, T_core_ of 12 ANs *increased significantly* from 36 ± 0.3°C to 37.2 ± 0.3°C. It was not clear whether this increase in T_core_ was related to a *decreased* TEF.

If a relationship between TEF and T_core_ could be determined, it would help explain whether this excess heat production may be fulfilling the biologic imperative of providing heat to assist with the re-establishment of T_core_, at least during the early phases of refeeding (Moukaddem et al., 1997; Russell et al., 2001; Marzola et al., 2013). If this proves to be the case, “heating” the AN during refeeding may be highly advantageous.

## Summary

It has been proposed that genetically prone ANs begin dieting and/or exercising, which results in weight loss. Weight loss initiates a chain of events resulting in a decrease in T_core_. In addition to the decrease in T_core_ (which cannot be directly monitored by the individual), the body’s periphery, primarily the hands, feels “uncomfortably” cold. ANs are now “motivated” to convert this sensation to one of “comfort.” Since ANs cannot activate the SNS to generate the required metabolic heat, they select the thermo-behavioral strategy of ExEx. Engagement in ExEx generates metabolic heat, which reduces discomfort in the hands, and importantly, assists in maintaining T_core_. Also associated with ExEx is the generation of brain neuromodulators, which “assist” with the perpetuation of ExEx and the subsequent activation of NEAT/SPA, suppression of appetite, anxiety reduction, and disrupted sleep/increased alertness.

**Figure 3:**
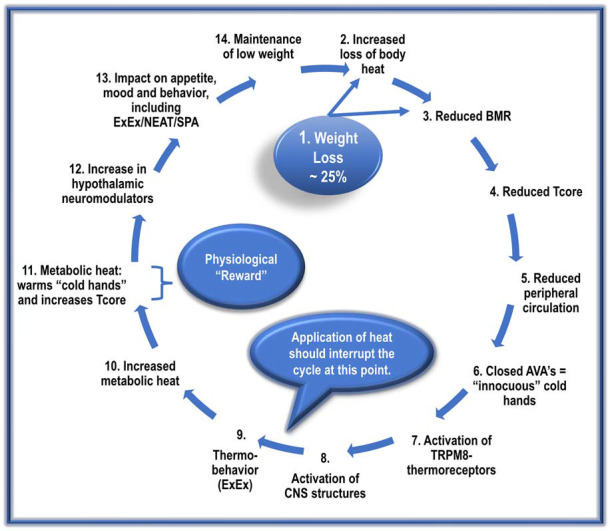
The proposed cyclic events, with repetition, manifest in the development and maintenance of AN.

Initially, the ANs may engage in ExEx to resolve their discomfort and “inadvertently,” but importantly, maintain T_core_. However, many additional *intrinsic rewards* (e.g., stimulation of dopaminergic system of the brain (Sodersten et al., 2016), as well as opioid peptides (Scheurink et al., 2010) as well as *extrinsic rewards* (e.g., compliments on weight loss), are associated with this condition; this encourages repetition. Repetition results in robust habit formation, making this condition highly intransigent (Figure 3; Uniacke et al., 2018).

The following sequence does not imply a once-off event. Instead, it suggests *cyclic repetitions* that gradually culminate in the manifestation of a full-blown AN condition.

1. Dieting and/or ExEx results in *dramatic weight loss* in genetically prone ANs.
2. Reduced vasoconstriction due to diminished SNS activity results in increased heat loss. Loss of fat mass (reduced insulation) further exacerbates heat loss.
3. A significant reduction in metabolic-related factors (reduced NE and T3) reduces BMR by about 20%. This lower BMR results in the production of less metabolic heat needed to maintain T_core_.
4. The increased loss of body heat and reduced BMR-heat availability results in an adaptive resetting of T_core_ to a lower level, possibly ~35–36.5°C.
5. Reduced SNS activity also inhibits activation of cardiovascular factors such as BP; this, in turn, *reduces peripheral circulation*, resulting in less blood-borne heat delivered to the periphery/hands.
6. The reduced peripheral circulation results in the closure of AVAs in the hands’ acral skin, *lowering hand skin temperature*.
7. The low hand temperatures induce the sensation of “thermal discomfort/innocuous cold” and activates local *TRPM8 thermoreceptors*.
8. The AN patient registers the “sensation” of “cold-hand-discomfort” in relevant CNS structures.
9. The AN patient is motivated to “select” a behavioral strategy to reduce the sensation of “cold hands.” The most frequently used and most effective strategy for ANs involves ExEx.
  (*If heat is made available, it should begin to interrupt the cycle at this point. That is, it should reduce the execution of ExEx)*.
10. The engagement by the ANs in ExEx increases the availability of metabolic heat.
11. With increased metabolic heat, there is an increase in hands’ perfusion. Increased perfusion with warm blood transforms the sensation of “cold-hand-discomfort” to one of “hand-comfort,” and simultaneously*, but more importantly*, assists with the maintenance of set-point T_core_.
12. The increase in *ExEx, together with caloric restriction*, increases brain levels of many central neuromodulators, including Uro II and III, ligands for CRH-R2, and Orexin-A.
13. Increased levels of these hypothalamic neuromodulators:
  - increase/maintain ExEx/NEAT/SPA.
  - decrease appetite/caloric ingestion.
  - increase anxiolysis.
  - disrupt sleep/increase alertness.
  - activate the brain reward system.
14. These factors contribute to the preservation of low weight and the perpetuation of this vicious cycle.

Hopefully, this hypothesis will generate research to further explore the role of hypothermia and hyperactivity in AN. “The success of treatment…depends on the correct diagnosis and a sufficient understanding of the factors underlying the symptom constellation of the disease or disorder” (Casper, 2018).

## Data Availability Statement

The original contributions presented in the study are included in the article, further inquiries can be directed to the corresponding author.

## Author Contributions

The author confirms being the sole contributor of this work and has approved it for publication.

## Conflict of Interest

The author declares that the research was conducted in the absence of any commercial or financial relationships that could be construed as a potential conflict of interest.

## Publisher’s Note

All claims expressed in this article are solely those of the authors and do not necessarily represent those of their affiliated organizations, or those of the publisher, the editors and the reviewers. Any product that may be evaluated in this article, or claim that may be made by its manufacturer, is not guaranteed or endorsed by the publisher.
